# Michigan Ways

**Published:** 1901-04

**Authors:** 


### MICHIGAN WAYS.

ANOTHER effort to strengthen the legislation regulating the practice of veterinary science has failed.

Some of Michigan's more prominent veterinarians have obtained their naturalization papers and will become eligible to register under the State law.

Why do not the discordant elements in Michigan get together and move in the way of higher veterinary education? To maintain a useless warfare because each cannot have its own way is harmful to the profession. Any law for the ultimate welfare of the State and the profession may work temporary injury or difficulties to that State or neighboring country, but it will inure in the end to the good of all. Stop trying to agree to disagree.

The Grand Rapids College should go to a three years' curriculum and force the issue on these lines over the State. The faculty and trustees should at once determine upon this step.

---

It was good to see the unbroken line of the old guard in Pennsylvania—Drs. John B., James B., Thomas B., and George B. Rayner, J. C. Foelker, N. Rectenwald, P. M. Minster, and A. R. May—among those on the benches of the association hall, at the meeting of the Pennsylvania State Veterinary Medical Association.

